# Confronting the global obesity epidemic: investigating the role and underlying mechanisms of vitamin D in metabolic syndrome management

**DOI:** 10.3389/fnut.2024.1416344

**Published:** 2024-08-09

**Authors:** Zihui Liang, Ziliang Wang, Xueyong Liu, Yu He

**Affiliations:** ^1^Department of Rehabilitation, Shengjing Hospital of China Medical University, Shenyang, Liaoning, China; ^2^Department of Physical Medicine and Rehabilitation, The Second Clinical College, China Medical University, Shenyang, Liaoning, China

**Keywords:** metabolic syndrome, vitamin D, insulin sensitivity, lipid metabolism, inflammation, immune response, therapeutic strategies

## Abstract

The escalating prevalence of MetS, driven by global obesity trends, underscores the urgent need for innovative therapeutic strategies. To gain a deeper understanding of the therapeutic potential of vitamin D in addressing MetS, we embarked on a targeted literature review that thoroughly examines the scientific underpinnings and pivotal discoveries derived from pertinent studies, aiming to unravel the intricate mechanisms through which vitamin D exerts its effects on MetS and its components. This article explores the multifunctional role of vitamin D in the management of MetS, focusing on its regulatory effects on insulin sensitivity, lipid metabolism, inflammation, and immune response. Through an extensive review of current research, we unveil the complex mechanisms by which vitamin D influences MetS components, highlighting its potential as a therapeutic agent. Our analysis reveals that vitamin D's efficacy extends beyond bone health to include significant impacts on cellular and molecular pathways critical to MetS. We advocate for further research to optimize vitamin D supplementation as a component of precision medicine for MetS, considering the safety concerns related to dosage and long-term use.

## 1 Introduction

In the wake of an accelerating modern lifestyle and shifting dietary patterns, obesity has ascended as a paramount challenge within the global public health sphere (1–3). Serving as a pivotal risk factor for MetS, obesity intricately intertwines with an array of metabolic aberrations, notably abnormal glucose metabolism, hypertension, and lipid dysregulation (4). This syndrome, distinguished by its prevalent occurrence and significant health ramifications, has become a critical focus in medical research endeavors. The prevalence of MetS exhibits considerable geographical variance, with rates spanning from 24.3% to 44.2% across specific regions in Asia, Europe, Mexico, and the United States (5). Notably, the prevalence in the United States has escalated from 36.2% to 47.3%, signaling a decline in cardiovascular health intricately linked to obesity, glucose imbalances, and blood pressure complications (6).

Amidst the exploration of metabolic syndrome, the significance of vitamin D has emerged with increasing prominence. This lipophilic vitamin, traditionally recognized for its pivotal roles in bone health and calcium-phosphorus homeostasis, has been unveiled by contemporary research to harbor extensive functionalities in immune modulation, cellular proliferation and differentiation, alongside energy metabolism (7–9). Specifically, within the milieu of obesity and metabolic syndrome, a deficiency in vitamin D is acknowledged as a consequential risk element (10–12).

Despite the widespread attention on vitamin D, its efficacy as a therapeutic agent remains inconsistent. Studies have shown that vitamin D supplementation may not necessarily reduce the risk of fractures or chronic diseases, and there is still debate about its optimal intake and supplementation methods. Therefore, comprehensive consideration of various factors is required to provide precise treatment recommendations for patients.

Given the rising prevalence of metabolic syndrome and its association with obesity, it is imperative to investigate potential therapeutic avenues. One such avenue that has garnered increasing attention is the role of vitamin D. This investigation aims to comprehensively explore the role of vitamin D in the therapeutic management of MetS, elucidating its multifaceted impact on MetS components, potential mechanistic pathways, and practical challenges in clinical implementation, with the ultimate goal of establishing a robust theoretical foundation for precision medicine and tailored therapeutic strategies targeting MetS.

## 2 Metabolic syndrome

Metabolic syndrome, characterized by multiple metabolic aberrations, has been witnessing a persistent global increase in prevalence, significantly impacting public health (5, 13–15). This intricate condition is driven by a synergy of adverse lifestyle choices, environmental factors, and genetic predispositions, with a notably higher incidence in developed nations (16–18). Concurrent with economic progress, the prevalence in developing countries is also on the rise. For instance, in China, with the rapid economic growth and urbanization over the past decades, there has been a significant increase in the prevalence of metabolic syndrome, particularly in urban areas where lifestyle changes such as sedentary work, westernized diet, and reduced physical activity have become more prevalent (19–21). The syndrome is intimately linked with the global rise in obesity, hypertension, diabetes, and other metabolic disorders, signaling an ongoing escalation in future disease risk (22, 23). Therefore, a thorough investigation into its risk factors and complications is imperative for devising effective prevention and control strategies.

## 3 Vitamin D

Vitamin D is a fat-soluble vitamin essential for human health (24–26). It serves as a precursor for several metabolites, including 25-hydroxyvitamin D3 [25-(OH)D3] and 1,25-dihydroxyvitamin D3 [1,25-(OH)2D3] (27). The human body cannot synthesize vitamin D on its own and must obtain it through sunlight exposure or dietary intake. Under the influence of ultraviolet radiation on the skin, 7-dehydrocholesterol is converted into a vitamin D precursor, which is then hydroxylated in the liver to form 25-hydroxyvitamin D [25-(OH)D]; this form of vitamin D is transported to the kidneys, where it is converted by the 1α-hydroxylase enzyme into the more active form, 1,25-dihydroxyvitamin D3 [1,25(OH)2D3] (28). 1,25(OH)2D3 exerts its biological effects by binding to the Vitamin D Receptor (VDR) (29, 30). Understanding this metabolic process leads us to further explore the crucial roles that vitamin D plays in the body. Not only is it pivotal in maintaining calcium-phosphorus metabolism balance and promoting bone health, but it also regulates immune functions, highlighting its multifaceted importance in human health (31–33).

## 4 Vitamin D and metabolic syndrome

Vitamin D plays a pivotal role in the prevention and management of MetS, a cluster of metabolic abnormalities that significantly impact public health. Its multifaceted functions extend beyond bone health, encompassing insulin sensitivity, lipid metabolism, inflammation reduction, and immune regulation, all of which are crucial in MetS prevention. Studies have elucidated a close correlation between vitamin D levels and the risk of metabolic syndrome (34–36). Insufficiency of vitamin D may elevate the incidence of metabolic syndrome, a pathological state encompassing obesity, hypertension, hyperglycemia, and dyslipidemia (37, 38). Vitamin D, through its regulation of calcium-phosphorus metabolism and maintenance of immune function, exhibits a spectrum of biological activities crucial for health (31–33). A deficiency in vitamin D can exacerbate insulin resistance, promote lipogenesis and inflammatory responses, thereby augmenting the risk of metabolic syndrome (39). Moreover, vitamin D influences cell signaling through the regulation of calcium ion balance, closely associating with the development of metabolic syndrome (40). Epidemiological studies support the relationship between low levels of vitamin D and an increased risk of metabolic syndrome, indicating that vitamin D supplementation could mitigate its prevalence (41–43).

**Figure 1:**
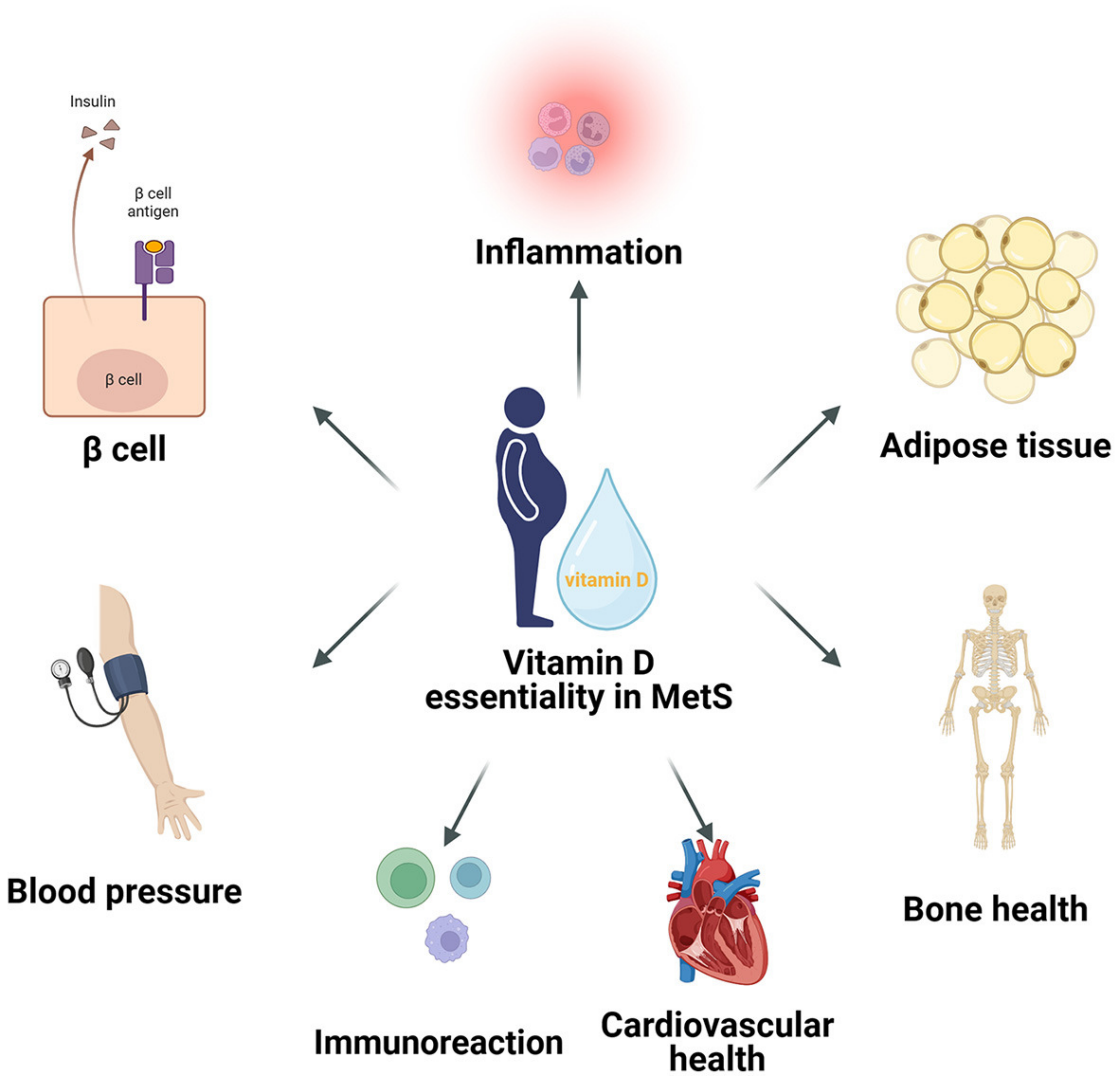
The critical roles of Vitamin D in metabolic syndrome, encompassing its significant impact on insulin sensitivity, lipid metabolism, inflammatory reduction, immune response, blood pressure regulation, cardiovascular health, and bone health.

Vitamin D plays a significant role in key areas of metabolic syndrome, including insulin sensitivity, lipid metabolism, inflammation reduction, immune response, blood pressure regulation, cardiovascular health, and bone health (Figure 1) (31, 34, 39, 43–46). In terms of insulin sensitivity, vitamin D enhances the binding capacity of insulin to its receptor and improves insulin efficacy by maintaining intracellular calcium ion balance. Regarding lipid metabolism, vitamin D modulates gene expression within adipocytes to reduce lipogenesis, promote lipolysis, and affect lipid transport in the blood, thereby lowering the risk of atherosclerosis. In reducing inflammatory responses, vitamin D inhibits the expression of inflammatory markers through certain signaling pathways, exhibiting anti-inflammatory effects, with its deficiency linked to the onset of various inflammatory diseases. In regulating immune responses, vitamin D, in conjunction with its receptor VDR, regulates the expression of a series of genes and proteins related to immune responses, thereby finely controlling immune reactions, inhibiting excessive activation and inflammation, and maintaining the balance, homeostasis, and health of the immune system, providing effective immune protection for the body. In blood pressure regulation, vitamin D affects the renin-angiotensin system and vascular smooth muscle function, contributing to the maintenance of normal blood pressure. The cumulative effect of these actions helps improve symptoms of metabolic syndrome and reduce the risk of related complications. In regulating cardiovascular health, vitamin D enhances intracellular substance synthesis, promotes vasodilation and anti-inflammation, and reduces the risk of atherosclerosis through cell signaling and gene regulation, ensuring the integrity of vascular structure and function. Additionally, vitamin D is crucial for bone health, enhancing the synthesis of bone matrix proteins and inhibiting bone resorption, while regulating gene transcription to promote calcium absorption and bone mineralization, and also inhibiting inflammation, reducing the risk of osteoporosis and fractures in patients with metabolic syndrome.

Although epidemiological studies suggest a link between low vitamin D levels and an elevated risk of metabolic syndrome, limitations such as the observational nature of these studies, population heterogeneity, and the complexity of metabolic syndrome itself hinder the establishment of causal relationships (47, 48). Rigorous clinical trials are needed to further investigate the potential benefits of vitamin D supplementation in mitigating metabolic syndrome symptoms.

## 5 Mechanisms of vitamin D action

### 5.1 Effects of vitamin D on metabolic pathways

Table 1 has summarized the molecular pathways of vitamin D's effects on metabolic syndrome-related mechanisms.

**Table 1:** Molecular pathways of vitamin D's effects on metabolic syndrome-related mechanisms.

**Mechanism type**	**Specific pathway/process**	**Related genes/proteins**	**References**
Insulin sensitivity	Regulation of insulin receptor expression and intracellular calcium ion balance	INSR, GLUT4, Ca^2+^	(49–51)
Lipid metabolism	Modulation of gene expression related to lipogenesis and lipolysis	FASN, UCP, C/EBPα, PPAR-γ	(52–54)
Inflammation and immune regulation	Inhibition of pro-inflammatory cytokine gene expression, regulation of immune cell differentiation	TNF-α, IL-6, IL-1β, NF-κB	(55–57)
Blood pressure regulation	Regulation of nitric oxide release from endothelial cells, influence on renin-angiotensin system	NO, RAS	(58–60)
Cardiovascular health	Promotion of endothelial nitric oxide synthase expression, reduction of oxidative stress	eNOS, NO, SOD	(61–63)
Bone health	Enhancement of bone matrix protein synthesis, inhibition of osteoclast activity	Osteocalcin, RANKL, OPG	(64–66)

#### 5.1.1 Regulation of insulin resistance and glucose metabolism by vitamin D

Vitamin D plays a crucial role in regulating insulin resistance and its associated processes (67, 68). This regulatory function is manifested at multiple levels; it can control gene transcription and cell signaling through various mechanisms, thereby alleviating insulin resistance, especially in adipose tissue (69). Vitamin D can increase the expression of insulin receptors, which is essential for maintaining insulin signaling (70). A deficiency in vitamin D can lead to a decrease in the number of insulin receptors, thereby inducing insulin resistance (71). Additionally, vitamin D deficiency may also cause an increase in intracellular Ca2+ concentration, reducing the activity of glucose transporter 4 (GLUT4), which is another key mechanism of insulin resistance (49). Inflammation in adipose tissue is also a critical factor in insulin resistance, primarily driven by adipose macrophages (72). Cytokines released by these macrophages, such as IL-6 and TNF-α, play significant roles in the development and progression of insulin resistance (50, 51). These cytokines can activate specific signaling pathways, such as Jun N-terminal kinase 1 (JNK1) and IKK-β/NF-κB, whose activation further leads to a reduction in insulin signaling (73). Importantly, vitamin D has anti-inflammatory effects, capable of reducing the inflammatory process by decreasing the release of chemokines and cytokines that drive inflammation and lowering the chemotaxis of monocytes (74). Vitamin D enhances insulin sensitivity by increasing insulin receptor expression and improving intracellular calcium ion balance. This leads to more efficient glucose uptake and utilization, thereby reducing the risk of developing type 2 diabetes, a key component of MetS.

Although vitamin D plays a significant role in regulating insulin resistance and related processes, there are contradictory findings regarding its direct effects on pancreatic β-cells and glucose metabolism (75, 76). Some studies indicate that vitamin D can directly influence β-cell function, enhance insulin secretion, and improve glucose metabolism, while other studies have not observed significant effects or have found inconsistent results. These discrepancies may stem from differences in experimental design and other variables. Therefore, future research needs to further explore the direct mechanisms of vitamin D's action on pancreatic β-cells, as well as how these mechanisms manifest in different populations.

#### 5.1.2 Regulation of fat metabolism by vitamin D

**Figure 2:**
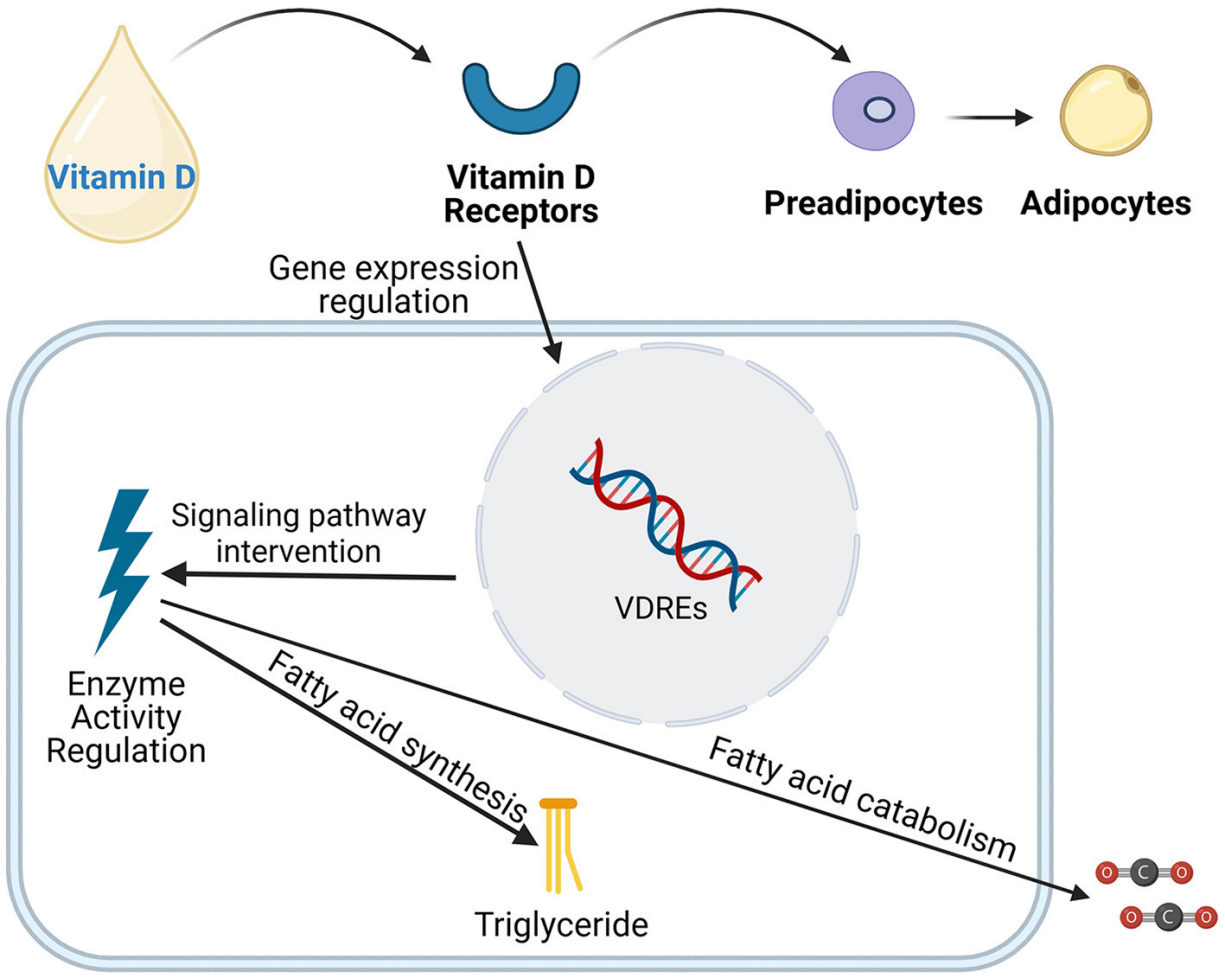
Vitamin D influences the differentiation, development of adipose cells, as well as the synthesis and breakdown of fatty acids through various mechanisms, including the regulation of gene expression, intervention in signaling pathways, and modulation of enzyme activity.

The mechanisms by which vitamin D acts in adipose tissue through the VDR receptor gene are complex (Figure 2). These findings collectively reveal vitamin D's multifaceted role in adipose tissue metabolism, including the regulation of lipolysis, fatty acid oxidation, and adipocyte differentiation (52, 77, 78). *In vitro* studies suggest that vitamin D can influence the fate of preadipocytes by regulating the expression of FASN (53), indicating its potential role in adipogenesis. Additionally, the vitamin D-VDR complex significantly impacts the differentiation process of adipocytes (79). Animal studies have shown that mice with a knockout of the VDR receptor gene (VDR-/-) exhibit an increased rate of energy metabolism, accompanied by an increase in the expression of the uncoupling protein (UCP) family in the mitochondrial respiratory chain and enhanced capacity for fatty acid β-oxidation, leading to a significant reduction in adipose tissue (80). Conversely, findings in VDR transgenic mice have been conflicting. While some studies show that overexpression of VDR in adipose tissue inhibits lipolysis and fatty acid β-oxidation, thereby inducing obesity (81), other studies may present contrasting results. This highlights the need for further research to clarify the exact role of VDR in adiposity. *In vitro* experiments further confirm that vitamin D can downregulate the expression of FASN, thereby inhibiting the transformation of pre-adipocytes (3T3-L1) into mature adipocytes (54). This finding reveals that vitamin D, by regulating the expression of key transcription factors, determines the direction of cell differentiation, playing a crucial role in forming different types of adipose tissue, such as white adipose tissue (WAT), brown adipose tissue (BAT), and beige adipose tissue (77). In adipose tissue, vitamin D can inhibit the expression of key transcription factors such as C/EBPα and PPAR-γ, thereby inhibiting fat construction (82). Additionally, vitamin D can influence lipid formation by activating C/EBPβ and ETO, inhibiting the transcription of C/EBPβ (31), and affecting adipocyte differentiation through the WNT/β-catenin signaling pathway (83). Moreover, research by Katayama and others found that VDR is also overexpressed in the sympathetic ganglia, adrenal medulla, and certain neurons in the central nervous system (84). These tissues are potential sites that could affect energy homeostasis, suggesting that vitamin D may influence energy homeostasis not only through its effects on adipose tissue but also through actions within the central nervous system affecting energy metabolism. By modulating gene expression within adipocytes, vitamin D promotes lipolysis and reduces lipogenesis, ultimately lowering plasma triglyceride and cholesterol levels. This reduces the risk of atherosclerosis and other cardiovascular complications associated with MetS.

#### 5.1.3 Anti-inflammatory action of vitamin D

Upon binding to the Vitamin D Receptor (VDR), active vitamin D undergoes a series of biochemical reactions, including phosphorylation, to form a heterodimer with the Retinoid X Receptor (RXR) and bind to VDREs to form a complex (85). This complex can influence gene transcription, thereby exerting anti-inflammatory and immunoregulatory biological functions (86). In terms of immunoregulation, vitamin D can affect the activity of immune cells such as antigen-presenting cells, monocytes-macrophages, and T and B lymphocytes (87, 88). However, it's important to note that the outcomes of vitamin D supplementation on inflammation and immune response can vary depending on the dosage, duration of supplementation, and the population studied (89). For instance, while appropriate levels of vitamin D can effectively modulate the immune system, excessive intake may lead to hypercalcemia and other adverse effects, which could potentially exacerbate inflammatory conditions (90). Active vitamin D can specifically activate peripheral CD4+ T cells, inhibiting the proliferation of Th1 cells and their production of pro-inflammatory cytokines, and stimulating Th2 cells to secrete IL-4, IL-5, and IL-10, making the immune tolerance response dominated by Th2 cells prevail over the immune rejection response dominated by Th1 cells (55). Additionally, vitamin D can downregulate the expression of Class II Major Histocompatibility Complex (MHC II), inhibit the maturation of dendritic cells, making it difficult for T cells to be activated and present antigens, thereby inducing immune tolerance (91). Simultaneously, vitamin D can inhibit the activation of B cells, induce the production of regulatory T cells (Treg), and inhibit the activity of Th17 cells. These mechanisms collectively enable vitamin D to play a significant role in anti-inflammation and immunoregulation (92). Moreover, it can inhibit the expression of inflammatory markers through the NF-κB classical inflammation signaling pathway or the p38 MAPK signaling pathway, exerting its anti-inflammatory effects (56). This may explain why vitamin D deficiency is associated with the incidence of inflammatory diseases such as obesity, metabolic syndrome, and type 2 diabetes. Conversely, excessive vitamin D intake, especially in the form of megadoses, has been reported to cause hypercalcemia, which can lead to calcification of soft tissues and potentially exacerbate inflammatory processes (90). Maintaining optimal vitamin D levels within the recommended dietary allowances is crucial to ensure its beneficial effects on inflammation and immune modulation. Vitamin D exerts potent anti-inflammatory effects by inhibiting the production of inflammatory cytokines, by macrophages and other immune cells. This attenuation of inflammation is pivotal in preventing the development of insulin resistance and other inflammatory-mediated metabolic disorders.

#### 5.1.4 Regulation of immune cells and immune responses by vitamin D

Vitamin D plays a pivotal role in immunoregulation, a function closely related to its specific metabolic pathways. Some 25-hydroxyvitamin D3 can also be metabolized into 24,25-dihydroxyvitamin D3 under the action of 24-hydroxylase (93), ensuring vitamin D's effective regulatory role in the immune system. Specifically, vitamin D can finely modulate the Th1/Th2 balance, inhibit the overproliferation of Th1 cells, and limit the secretion of Th1-type cytokines, including IFN-γ, IL-2, and TNF-α, thus preventing excessive activation of autoimmune responses (57). Additionally, vitamin D can block the activation and secretion of the key autocrine growth factor IL-2 in T lymphocytes, further reducing the risk of autoimmune responses (94). Moreover, recent studies have shown that the immune response to vitamin D can vary among different populations due to genetic, environmental, and lifestyle factors. For instance, certain ethnic groups may have different vitamin D receptor polymorphisms, affecting their response to vitamin D and subsequently, their immune reactions (95). Therefore, it is crucial to consider population-specific differences when evaluating the immunomodulatory effects of vitamin D. Vitamin D also induces the polarization of CD4+ T lymphocytes toward a Th2 response, promoting the secretion of Th2-type cytokines (such as IL-4, IL-5, IL-9, IL-10, and IL-13), inhibiting excessive inflammatory responses, and maintaining the balance of the immune system (96). Mariz et al. confirmed that in female groups with higher levels of vitamin D, a lower mean concentration of TNF-α in the serum was observed, providing strong evidence of the significant role of vitamin D in immunoregulation (97). Furthermore, vitamin D deficiency has been linked to an increased risk of autoimmune diseases, particularly those related to MetS. Studies suggest that optimizing vitamin D levels may help reduce the severity and progression of these conditions (98). These findings not only reveal the close link between vitamin D metabolism and immunoregulation but also suggest potential applications of vitamin D in the treatment of immune-related diseases, providing a theoretical basis for such use.

#### 5.1.5 Vitamin D and its role in health issues associated with metabolic syndrome

##### 5.1.5.1 Blood pressure regulation

Vitamin D plays a pivotal role in blood pressure regulation, particularly in the context of metabolic syndrome. Its active form, 1,25-dihydroxyvitamin D3, regulates the expression of genes involved in blood pressure control, including those encoding renin and angiotensinogen (7, 99). By inhibiting renin synthesis, vitamin D reduces the production of Angiotensin II, a vasoconstrictor that increases peripheral vascular resistance. This leads to decreased vasoconstriction and lower peripheral vascular resistance, effectively regulating blood pressure (58). Additionally, vitamin D promotes nitric oxide release from endothelial cells, a potent vasodilator, maintaining homeostasis and normal blood flow (59). Its antioxidant and anti-inflammatory properties reduce oxidative stress and inflammatory responses that damage blood vessels, further contributing to blood pressure reduction (60). Vitamin D not only regulates blood pressure through mechanisms such as inhibiting renin synthesis, promoting nitric oxide release, and reducing oxidative stress and inflammation, but its overall therapeutic effects on these processes also contribute to the prevention and management of metabolic syndrome (100).

##### 5.1.5.2 Cardiovascular health

In the context of metabolic syndrome, vitamin D exerts multifaceted effects on cardiovascular health. It maintains serum calcium levels by regulating calcium balance and bone metabolism, involving interactions with parathyroid hormone and renin (61, 101). Vitamin D directly protects the cardiovascular system by inhibiting inflammatory responses and oxidative stress, improving insulin resistance and glucose metabolism (86, 102). It also regulates endothelial function, lipid metabolism, and reduces arterial calcification (62, 103, 104), critical for maintaining cardiovascular health. By activating intracellular signaling pathways, vitamin D fine-tunes the expression of genes related to cell proliferation, migration, and apoptosis, preventing atherosclerosis development (63, 105). Finally, vitamin D interacts with other nutrients, jointly maintaining normal cardiovascular function (106). In the context of metabolic syndrome, vitamin D exerts multifaceted therapeutic effects on cardiovascular health, including maintaining calcium balance, protecting against inflammatory responses and oxidative stress, regulating endothelial function, reducing arterial calcification, and preventing atherosclerosis development, all of which contribute to the prevention and management of metabolic syndrome.

##### 5.1.5.3 Bone health

Vitamin D is a key regulatory factor for bone health, especially in the context of metabolic syndrome. Its active form forms a complex with the VDR, regulating bone mineralization (64, 65). This regulation is crucial for maintaining bone health in MetS, where bone metabolism may be altered (107, 108). Vitamin D ensures an adequate supply of calcium and phosphate for normal mineralization by regulating their intestinal absorption (66). It also regulates osteoprotegerin, a key factor in bone formation, mineralization, and resorption (109, 110). By stimulating the expression of bone cell-specific genes, vitamin D promotes the synthesis of sclerostin, further contributing to bone health maintenance (111). Additionally, vitamin D modulates the immune system, inhibiting inflammatory cytokine production, reducing bone inflammation, and lowering the risk of osteoporosis and fractures (112–114). Vitamin D exerts therapeutic effects on bone health in the context of metabolic syndrome, regulating bone mineralization, ensuring adequate calcium and phosphate supply, modulating osteoprotegerin levels, promoting sclerostin synthesis, and modulating the immune system to reduce inflammation and lower the risk of osteoporosis and fractures, all contributing to the prevention and management of metabolic syndrome.

Furthermore, future studies should delve deeper into the interactions between vitamin D supplementation and other metabolic syndrome (MetS) treatments. Understanding these interactions is crucial for optimizing therapeutic approaches, enhancing treatment efficacy, and ensuring patient safety. By considering the synergistic or antagonistic effects of vitamin D with other medications, we can provide more personalized and precise treatment strategies for patients.

### 5.2 Cellular and molecular mechanisms of vitamin D

#### 5.2.1 Vitamin D's multifaceted roles in pancreatic β-cell function and glucose regulation: a deep dive into the mechanisms

Vitamin D holds a pivotal position in pancreatic β-cell function and glucose regulation (115). Its direct interaction with β-cells at the cellular level enhances insulin gene expression, thereby promoting insulin synthesis and release (116). These processes are crucial for maintaining blood glucose homeostasis (117).

##### 5.2.1.1 Vitamin D and the VDR

When vitamin D binds to the VDR, it triggers a cascade of signaling pathways (118, 119). These pathways not only regulate insulin production but also precisely adjust the expression of genes associated with apoptosis. This fine-tuning supports β-cell survival and ensures their optimal function (120, 121).

##### 5.2.1.2 Molecular mechanisms of vitamin D action

Digging deeper, the formation of the vitamin D-VDR complex enables the recognition of Vitamin D Response Elements (VDREs) located within the cell nucleus. This recognition mechanism regulates the transcription of genes closely linked to glucose metabolism and β-cell apoptosis (86). Beyond this, vitamin D also contributes to maintaining cellular homeostasis in β-cells. It achieves this by adjusting calcium ion concentrations, thus preventing calcium overload-induced apoptosis. Additionally, it bolsters the cells' antioxidant defenses against oxidative stress (122, 123).

##### 5.2.1.3 Practical and clinical implications: harnessing vitamin D for improved type 2 diabetes management

Vitamin D plays a pivotal role in pancreatic β-cell function and glucose regulation. It not only promotes insulin synthesis and release, thereby maintaining glucose homeostasis, but also safeguards β-cells against damage by regulating intracellular calcium ion balance and enhancing antioxidant defenses, prolonging their survival and function. This understanding offers novel therapeutic avenues for managing type 2 diabetes, wherein personalized vitamin D supplementation strategies, as adjunctive therapy, may contribute to reducing the need for exogenous insulin and improving glycemic control in patients (124).

##### 5.2.1.4 Challenges, limitations, and future directions

Despite the immense potential of vitamin D in modulating immune function and pancreatic β-cell health, determining the optimal dosage and supplementation approach remains challenging (125). Variations in individual responses to vitamin D necessitate consideration of personal differences in therapeutic strategies to achieve personalized supplementation. Additionally, the safety and potential side effects of long-term vitamin D supplementation warrant further evaluation (126).

Current research predominantly relies on observational studies and animal experiments, lacking large-scale, long-term clinical trials to validate the precise efficacy of vitamin D in diabetes management. Moreover, the incomplete understanding of vitamin D's interactions with other medications and nutrients may impact its application effectiveness in clinical practice.

Future research should prioritize high-quality randomized controlled trials to clarify the effectiveness and safety of vitamin D in diabetes management. Additionally, exploring the synergistic effects of vitamin D with other therapeutic approaches and developing optimized supplementation protocols tailored to different populations will be crucial for advancing its application in diabetes treatment. Furthermore, intensifying studies on the underlying mechanisms of vitamin D's actions can unveil more of its functions in diabetic pathogenesis.

#### 5.2.2 Vitamin D's role in adipocyte differentiation and metabolism: mechanisms and current understanding

Vitamin D plays a crucial role in the differentiation and metabolism of adipocytes, processes that are fundamental to adipose tissue formation and function (80).

##### 5.2.2.1 Adipocyte differentiation and vitamin D

During adipocyte differentiation, pre-adipocytes transform into mature adipocytes in response to specific environmental signals. Vitamin D, through its activation of the VDR, modulates this transition by precisely adjusting the expression of genes intricately involved in adipocyte differentiation (77). This regulatory action notably decelerates the differentiation process, ultimately leading to a reduction in the overall number of fat cells (53, 79).

##### 5.2.2.2 Vitamin D and adipocyte metabolism

Beyond its role in differentiation, vitamin D also regulates the metabolic functions of adipocytes (127). It achieves this by balancing the synthesis and breakdown of fatty acids within these cells. By modulating the activity of enzymes that are involved in fatty acid metabolism, vitamin D effectively decreases fatty acid synthesis and increases its oxidation, thereby reducing intracellular fat storage (128, 129).

##### 5.2.2.3 Molecular mechanisms

At the molecular level, the binding of vitamin D to VDR enables it to specifically recognize and interact with VDREs in the cell nucleus. This interaction regulates the transcription of genes directly implicated in fat formation (130). Through this mechanism, vitamin D selectively inhibits or activates specific genes, offering precise control over the rate and extent of adipogenesis (131). Research by Mutt et al. has shown that vitamin D can downregulate the expression of key genes, such as fatty acid synthase (FASN), which is essential for fatty acid synthesis (132). By suppressing these genes, vitamin D further diminishes fatty acid production, thus inhibiting adipogenesis. Moreover, vitamin D modulates the fat formation process by influencing intracellular signaling pathways, which are crucial for information transfer within cells (133). Harahap et al. suggest that vitamin D can activate or inhibit specific kinases and transcription factors, further regulating adipogenesis (134).

##### 5.2.2.4 Practical and clinical implications: optimizing vitamin D supplementation for weight and lipid control

Given the specific mechanisms by which vitamin D regulates adipocyte differentiation and metabolism, it holds significant practical implications for weight and lipid control in clinical settings. By precisely modulating the dosage of vitamin D supplementation, it can effectively decelerate the differentiation process of adipocytes, leading to a reduction in the total number of fat cells and contributing to weight management (135). Additionally, vitamin D's ability to balance fatty acid synthesis and oxidation, thereby decreasing intracellular fat storage, facilitates improved lipid profiles, specifically reducing triglycerides and low-density lipoprotein (LDL) cholesterol levels while potentially increasing high-density lipoprotein (HDL) cholesterol (136). As such, for patients with obesity and metabolic syndrome, individualized vitamin D supplementation protocols may serve as an adjunct approach to weight control, enhancing the effectiveness of lipid management and mitigating the risk of cardiovascular diseases. Future research and clinical practice should delve deeper into determining optimal vitamin D doses for diverse populations, aiming to maximize its benefits in terms of weight and lipid control.

##### 5.2.2.5 Challenges, limitations, and future directions

In the exploration of vitamin D's therapeutic potential in metabolic syndrome management, several challenges and limitations have emerged. Despite the widely recognized significance of vitamin D in adipocyte growth and metabolism, there remains a lack of consensus on the optimal daily dose of vitamin D supplementation (137). This is primarily due to the inconsistent findings from different studies, which can be attributed to variations in experimental conditions and test populations. Moreover, the potential interaction between vitamin D and other nutrients adds to the complexity of devising effective supplementation strategies (138).

Additionally, individual differences in response to vitamin D underscore the need for personalized supplementation approaches. Given these uncertainties, further research is imperative to determine the optimal individualized dose and method of vitamin D supplementation for patients with metabolic syndrome.

Looking ahead, future research directions should aim to clarify the mechanisms underlying vitamin D's multifaceted effects, explore its interactions with other hormones and nutrients, and assess the long-term safety and efficacy of supplementation. By addressing these challenges and limitations, we can develop more targeted and effective therapeutic strategies for the management of metabolic syndrome.

#### 5.2.3 Vitamin D's role in immune regulation and inflammation: mechanisms and current understanding

Vitamin D profoundly influences the development and functional expression of immune cells (139, 140). It meticulously adjusts the gene expression profiles of key inflammatory regulators, such as macrophages, T cells, and B cells, by activating the VDR within these cells. This, in turn, regulates the inflammatory mediators they release.

##### 5.2.3.1 Vitamin D and immune cell gene expression

Upon binding to VDR, vitamin D forms a complex that can precisely identify and bind to VDREs in the cell nucleus (141). This binding regulates the transcription of genes closely related to inflammation, providing a molecular basis for the immune response modulation (142, 143). Specifically, vitamin D can inhibit the production of inflammatory cytokines, such as TNF-α, IL-1β, and IL-6, by macrophages, effectively mitigating the body's inflammatory response (144).

##### 5.2.3.2 Regulation of inflammatory genes

The vitamin D-VDR complex selectively inhibits or activates specific inflammatory genes, offering a deeper understanding of how inflammation is controlled at the molecular level (145). Studies have confirmed that vitamin D can inhibit the activation of the key inflammatory transcription factor NF-κB, subsequently downregulating the expression of various inflammatory genes and reducing the production of inflammatory mediators (56).

##### 5.2.3.3 Intracellular signaling pathways

Beyond direct gene regulation, vitamin D also influences the inflammatory process by regulating intracellular signaling pathways (146). These pathways, akin to information superhighways within cells, are effectively modulated by vitamin D, adjusting the synthesis and release of inflammatory mediators (147). Specifically, vitamin D can activate signaling molecules such as PKA and PKC, further regulating downstream inflammatory responses (70, 148, 149).

##### 5.2.3.4 Practical and clinical implications of the study: vitamin D as an immunomodulator and anti-inflammatory agent

In clinical practice, vitamin D supplementation demonstrates remarkable immunomodulatory and anti-inflammatory potential (150). By inhibiting the release of pro-inflammatory cytokines and key transcription factors, vitamin D offers a promising therapeutic approach for alleviating systemic inflammation under conditions such as metabolic syndrome and autoimmune diseases (141). This mechanism not only targets the underlying inflammatory pathways but also addresses the broader spectrum of health complications associated with chronic inflammation, thereby enhancing overall patient outcomes and quality of life.

##### 5.2.3.5 Challenges, limitations, and future directions

Despite the immense potential of vitamin D in immune regulation and anti-inflammatory actions, its clinical application faces several challenges. The optimal dosage and duration of vitamin D supplementation remain unclear, necessitating further research (151). Moreover, the incomplete understanding of its interaction mechanisms with other nutrients and medications hinders its use in treating multifactorial diseases. Additionally, long-term excessive intake of vitamin D may pose potential health risks, such as hypercalcemia and soft tissue calcification, limiting its widespread clinical adoption.

Research on vitamin D's immune regulatory and anti-inflammatory effects still has limitations. Most studies focus on animal models and *in vitro* cell cultures, lacking large-scale clinical trials to validate its efficacy (152). The complex mechanisms of vitamin D involve multiple signaling pathways and cell types, making it difficult to determine its precise role. Furthermore, individual variability in responses to vitamin D may be influenced by genetic polymorphisms and other genetic factors.

Future research should focus on several key areas. Large-scale clinical trials are needed to verify the role of vitamin D in preventing and treating metabolic syndrome and related inflammatory diseases. In-depth exploration of vitamin D's mechanisms, particularly in immune regulation and anti-inflammation, is essential. Moreover, novel strategies for personalizing vitamin D supplementation dosages and durations must be developed to maximize its therapeutic potential while mitigating potential health risks. Through these efforts, we can anticipate vitamin D playing a more significant role in immune modulation and anti-inflammation, offering new insights and approaches for treating metabolic syndrome and related inflammatory conditions.

#### 5.2.4 Roles of vitamin D in regulating other metabolic syndrome-related factors and research status

##### 5.2.4.1 Blood pressure regulation

Vitamin D plays a crucial role in blood pressure regulation by influencing the function of endothelial cells and vascular smooth muscle cells (153, 154). It promotes the release of nitric oxide (NO), a vasodilator, from endothelial cells, contributing to the maintenance of normal blood pressure levels (155). Additionally, vitamin D inhibits the proliferation and migration of vascular smooth muscle cells, enhancing vascular stability and elasticity, further reducing the risk of hypertension (156, 157). Research indicates that vitamin D modulates blood pressure dynamics by regulating the expression of genes associated with blood pressure regulation, such as those involved in the renin-angiotensin system (RAS) (158–160). Furthermore, vitamin D's regulation of intracellular calcium ion balance indirectly affects blood pressure homeostasis (161).

##### 5.2.4.2 Cardiovascular health

Vitamin D contributes significantly to cardiovascular health by enhancing the expression of nitric oxide synthase in cardiovascular cells, thereby promoting nitric oxide synthesis and release (162, 163). This aids in maintaining normal vascular function and elasticity, reducing the risk of cardiovascular diseases (164). Additionally, vitamin D activates intracellular signaling pathways in cardiovascular cells, fine-tuning the expression of genes and proteins related to cell proliferation, migration, and apoptosis (165). This inhibits abnormal proliferation and migration of vascular smooth muscle cells, effectively preventing the development of atherosclerosis. Vitamin D further mitigates oxidative stress and inflammatory responses in the cardiovascular system by regulating the expression of antioxidant and anti-inflammatory genes, thereby maintaining the normal structure and function of blood vessels (157, 166).

##### 5.2.4.3 Bone health

Vitamin D directly affects osteoblasts and osteoclasts, crucial cells for bone formation and remodeling (167). It enhances the synthesis of bone matrix proteins, such as osteocalcin and collagen, essential for bone framework construction (168, 169). Simultaneously, vitamin D inhibits osteoclast activity, reducing bone resorption and degradation, thus maintaining bone strength and stability (170). By regulating the expression of genes related to calcium absorption and transport, vitamin D ensures an adequate calcium supply for bones, promoting normal bone mineralization and growth (171, 172). Furthermore, vitamin D indirectly safeguards bone health by modulating the immune system, inhibiting inflammatory cytokine production, and reducing bone inflammation, thereby lowering the risk of osteoporosis and fractures (173, 174).

##### 5.2.4.4 Practical and clinical implications of the study

For blood pressure control, the crucial role of vitamin D in promoting the release of nitric oxide from endothelial cells and inhibiting the proliferation and migration of vascular smooth muscle cells highlights its potential as an adjuvant therapy in hypertension management (175). Regular supplementation of vitamin D could help maintain normal blood pressure levels and reduce the risk of hypertension, thereby improving cardiovascular outcomes.

In terms of cardiovascular health, the study underscores the importance of vitamin D in promoting nitric oxide synthesis and release, enhancing vascular function and elasticity (176). This suggests that vitamin D supplementation could aid in preventing cardiovascular diseases by improving the overall health of blood vessels. Moreover, vitamin D's ability to regulate intracellular signaling pathways related to cell proliferation, migration, and apoptosis in cardiovascular cells can contribute to the prevention of atherosclerosis.

Regarding bone health, the direct effects of vitamin D on osteoblasts and osteoclasts, as well as its role in regulating calcium metabolism, have important implications for the prevention and treatment of bone-related disorders. By ensuring adequate calcium supply for bones and inhibiting bone resorption, vitamin D supplementation can strengthen bone structure and reduce the risk of osteoporosis and fractures (177). This is particularly relevant for populations at risk of bone loss, such as the elderly or individuals with chronic diseases.

##### 5.2.4.5 Challenges, limitations, and future directions

Vitamin D's clinical use faces challenges. Individual variability in response necessitates tailored dosing. Complex interactions with other nutrients hinder precise supplementation. Factors like lifestyle, environment, and genetics yield inconsistent results (1).

Research limitations include the predominance of observational studies, sample heterogeneity, and limited data on long-term safety and effectiveness, particularly in diverse populations (178).

Randomized trials are needed to establish causality. Explore vitamin D-nutrient interactions for personalized supplementation. Investigate metabolic differences across populations to enhance results' generalizability. Monitor long-term safety and effectiveness for safe clinical application.

### 5.3 Interactions of vitamin D with other metabolic hormones

The mechanisms of interaction between vitamin D and other metabolic hormones are complex and crucial for maintaining normal physiological functions in the human body (179). Firstly, in the interaction between vitamin D and insulin, the mechanism is primarily manifested by the action of the active form of vitamin D, 1,25-dihydroxyvitamin D3, in the pancreas. It can promote β-cell synthesis and secretion of insulin, thereby regulating blood sugar levels (180). At the same time, vitamin D can inhibit pancreatic α-cells, reducing the secretion of glucagon and stabilizing blood sugar (181). Insulin also affects vitamin D levels by promoting intestinal absorption of vitamin D, increasing the body's vitamin D concentration (182). Their synergistic action is evident as vitamin D enhances the expression of insulin receptor genes, improving cell sensitivity to insulin, which helps in improving insulin resistance (183).

Secondly, the interaction mechanism between vitamin D and parathyroid hormone (PTH) is primarily reflected in maintaining calcium balance (184). When blood calcium levels drop, vitamin D stimulates the activation of the calcium-sensing receptor (CaSR) in the parathyroid gland, leading to an increase in PTH levels, which then promotes the kidney to synthesize 1,25(OH)2D, enhancing the kidney's reabsorption of calcium (185). Simultaneously, 1,25(OH)2D and PTH act together on osteoblasts, regulating bone metabolism processes. This mechanism ensures the body's priority in maintaining calcium balance but may also lead to bone health issues (186).

Furthermore, the interaction mechanism between vitamin D and cortisol is represented by a direct antagonistic action. Vitamin D promotes the absorption and utilization of calcium, while cortisol inhibits these processes (187). This antagonistic action helps maintain the body's calcium homeostasis and related physiological functions.

Lastly, in the interactions between vitamin D and sex hormones like estrogen and testosterone, the mechanism is mainly manifested in their impact on bone health (188). Vitamin D and estrogen jointly regulate the function of osteoblasts and osteoclasts, maintaining bone homeostasis (189). Vitamin D may influence the synthesis and receptor expression of testosterone, regulating the physiological processes of testosterone (190). These interaction mechanisms collectively maintain bone health and the balance of sex hormones (191).

## 6 Conclusion

Vitamin D plays a crucial role in the treatment of metabolic syndrome. It is not only vital for bone health and calcium-phosphate metabolism but also plays a role in immune regulation and energy metabolism. Epidemiological and clinical studies indicate that vitamin D deficiency is closely associated with obesity, insulin resistance, dyslipidemia, and hypertension, which are components of metabolic syndrome. Its mechanisms of action involve the regulation of key metabolic pathways and interactions with other hormones. Although there is controversy over the dosage and efficacy of vitamin D supplementation, its potential in the treatment of metabolic syndrome warrants further exploration. Considering the safety concerns associated with long-term excessive intake, future research needs to define the appropriate dosage of vitamin D supplementation and its long-term safety. In summary, research on vitamin D provides new perspectives and strategies for the prevention and treatment of metabolic syndrome.

## Author contributions

ZL: Writing – original draft, Conceptualization. ZW: Conceptualization, Supervision, Writing – original draft. YH: Conceptualization, Funding acquisition, Supervision, Writing – review & editing. XL: Conceptualization, Supervision, Writing – review & editing.
